# Defining human mesenchymal stem cell efficacy *in vivo*

**DOI:** 10.1186/1476-9255-7-51

**Published:** 2010-10-25

**Authors:** Tracey L Bonfield, Mary T Nolan (Koloze), Donald P Lennon, Arnold I Caplan

**Affiliations:** 1 Department of Pediatrics, Case Western Reserve University, Cleveland, OH. USA; 2 Skeletal Research Center, Department of Biology, Case Western Reserve University, Cleveland, OH. USA

## Abstract

Allogeneic human mesenchymal stem cells (hMSCs) can suppress graft versus host disease (GvHD) and have profound anti-inflammatory and regenerative capacity in stroke, infarct, spinal cord injury, meniscus regeneration, tendinitis, acute renal failure, and heart disease in human and animal models of disease. There is significant clinical hMSC variability in efficacy and the ultimate response *in vivo*. The challenge in hMSC based therapy is defining the efficacy of hMSC *in vivo*. Models which may provide insight into hMSC bioactivity *in vivo *would provide a means to distinguish hMSCs for clinical utility. hMSC function has been described as both regenerative and trophic through the production of bioactive factors. The regenerative component involves the multi-potentiality of hMSC progenitor differentiation. The secreted factors generated by the hMSCs are milieu and injury specific providing unique niches for responses *in vivo*. These bioactive factors are anti-scarring, angiogenic, anti-apoptotic as well as regenerative. Further, from an immunological standpoint, hMSC's can avoid host immune response, providing xenographic applications. To study the *in vivo *immuno-regulatory effectiveness of hMSCs, we used the ovalbumin challenge model of acute asthma. This is a quick 3 week *in vivo *pulmonary inflammation model with readily accessible ways of measuring effectiveness of hMSCs. Our data show that there is a direct correlation between the traditional ceramic cube score to hMSCs attenuation of cellular recruitment due to ovalbumin challenge. The results from these studies verify the *in vivo *immuno-modulator effectiveness of hMSCs and support the potential use of the ovalbumin model as an *in vivo *model of hMSC potency and efficacy. Our data also support future directions toward exploring hMSCs as an alternative therapeutic for the treatment of airway inflammation associated with asthma.

## Introduction

Human mesenchymal stem cells (hMSCs) from marrow reside *in situ *as pericytes that are hypothesized to function as sentinels to guard against self-surveillance by T-cells at sites of tissue damage [1]. The local titers of hMSCs depend on the vascular density at that site and on other factors. Although hMSCs were first thought to function as the source for cellular replacement therapies, their immuno-modulatory and trophic activities have the potential for profound therapeutic impact in diseases associated with sustained inflammation. By providing additional hMSCs through systemic routes, both immuno-regulatory and regenerative trophic activities at sites of inflammation and tissue damage can be enhanced [2].

hMSCs are non-hematopoietic, multi-potent progenitor cells, which have the ability to influence immune effector cell development, maturation and function as well as allo-reactive T-cell responses through the production of bioactive cytokines and proteins [3]. The designation of hMSCs is based upon extensive immunophenotyping using surface antigens and ability to function in *in vitro *models [4]. MSCs are immuno-modulatory and express no MHC class II, making hMSCs a viable therapeutic across tissue typing [5,6]. hMSCs produce large quantities of bioactive factors which provide molecular signatures for the pathway and activity status of the responding cells [7,8]. These bioactive factors are anti-scarring, angiogenic, anti-apoptotic and regenerative (i.e., mitotic for host-derived progenitor cells). As evidence of the profound effect of hMSCs on the immune system, our colleagues and others have reported that hMSCs are well tolerated and therapeutically active in immuno-competent rodent models of multiple sclerosis and stroke [9-11]. Thus, xenogenic hMSCs repress host immunological surveillance in rodents while at the same time producing reparative growth factors.

An important issue in hMSC biology has been understanding the significant variability among cell preparations. Models need to be developed which not only show the relative unique therapeutic application of hMSCs, but also measure *in vivo *function (i.e., therapeutic potency). Acute (short-term) *in vivo *models of inflammation have the potential to provide vehicles for hMSC efficacy assessment. Although chronic (long-terms) models provide valid options for study, our focus was on an *in vivo *model which would provide a quick answer of hMSC activity, with the clinical and therapeutic applications in mind. Culture-expanded hMSCs increase in size with each passage and thus, on a size basis alone are observed to lodge in the lungs in substantial numbers when given intravenously [12]. hMSCs have the potential to provide a local source of trophic factors in the pulmonary environment, which may result in changes in lung inflammation [13]. This circumstance allows us to determine the effects of hMSCs in the initiation of inflammatory lung diseases and to establish the criteria for efficacy of different donor hMSCs on early stages of asthma in rodent models.

Acute bronchial asthma has been characterized by allergic airway inflammation, which induces both cytological as well as histological changes in the airway structure over time [14]. The pathogenic characteristics of allergic asthma are associated with airway inflammation and infiltration of mast cells, basophils, eosinophils, monocytes and T helper type 2 lymphocytes, along with the production of isotype-specific immunoglobulin E (IgE) [15,16]. Several animal models have been developed to model human airway disease associated with acute asthma, which have the capacity to mimic the histological and pathologic changes in the lung. A commonly used model to study airway inflammation *in vivo *involves primary sensitization with ovalbumin (OVA) followed by daily intranasal challenge with the antigen to generate airway inflammation mimicking the acute asthma exacerbation. This short-term model can provide the basis for studying the trophic impact of lodged hMSCs on development of lung inflammation associated with acute asthma challenge and the potential benefit of hMSCs in circumventing acute asthmatic inflammatory disease.

The results of these studies provide the foundation for understanding the role of hMSCs in altering inflammatory processes *in vivo *and provide support for the utilization of the short-term acute asthma model as a validation tool for hMSC efficacy and function *in vivo*.

## Materials and methods

### Mouse Model

Balb/c mice were purchased from the Jackson Laboratories (Bar Harbor, Maine) at 5 weeks, allowed to adjust to environment for 1 week. On day 0 (6 weeks of age) mice were sensitized by intra-peritoneal injections (100 μL) of 10 μg of ovalbumin (OVA) emulsified in 1.5 mg of Al (OH)_3_. On day 14, mice were exposed to 1% wt/vol OVA [17,18] in sterile saline by aerosolization every day for 5 days. Sham sensitization and sham challenges were carried out with sterile saline. hMSCs were given on either day 14 or day 16 by tail vein injection with 1 × 10^6 ^hMSC/mouse in 100 ul of PBS. For each of a minimum of 3 experiments, 4-6 animals were evaluated for the following: saline challenge, saline challenge +hMSC, ovalbumin challenge, ovalbumin challenge+hMSCs. In addition, subsets of saline and ovalbumin mice were given bone marrow derived macrophages (BMDM) as a control for the hMSC. As a positive control for immunosuppression, a subset of the ovalbumin sensitized, ovalbumin challenged mice received dexamethasone at 10 mg/kg. In all cases, the dexamethasone significantly decreased total cell recruitment and eosinophil counts (data not shown) supporting our ability to measure changes in airway inflammation.

### Lung Inflammation

Mice were injected subcutaneously with ketamine (80 mg/kg) and xylazine (10 mg/kg). The thoracic cavity was opened and lungs exposed. Bronchoalveolar lavage (BAL) was performed by inserting a cannula through a cut in the trachea into the bronchi and infusing 3 × 1 ml aliquots of warm PBS containing 0.2% lidocaine. The BAL fluid sample was recovered by aspirating the liquid with a syringe. Cells were separated from lavage fluid and differential analysis was evaluated using cytospins and Wright-Giemsa staining. Remaining cells were frozen for future analysis. BAL fluid was frozen at -80°C until assessment for cytokines.

#### Lung Pathology

Lungs were either perfused with 10% formalin or snap frozen. Mean viability of lavage cells was > 95% by trypan blue dye exclusion. Animals were assessed for inflammation by BAL and a separate set of animals was evaluated for lung histology using H&E and trichrome staining.

#### Systemic Inflammation

Animals underwent cardiac puncture after completion of lavage and the blood was processed to obtain serum and plasma. Serum cytokines were measured using ELISAs or Luminex technology.

### MSCs

Cells were isolated from the marrow of healthy volunteers as described elsewhere [19]. Briefly, the marrow aspirates were layered on density gradients, and the light cell fraction was retrieved, washed and plated in selected batches of 10% fetal bovine serum (FBS) in Dulbecco's Modified Eagle Medium (DMEM) [20]. Within 14 days the colony-forming units were selectively expanded to near confluency and the cells were then lifted from the dish with trypsin and replated at 4500 cells/cm^2 ^to ensure they maintained active cell division. As determined by other studies, cells cultured using this protocol were homogenous when FAC-Sorted using over 100 different cell surface antibodies [4]. All 7 hMSCs preparations were harvested from culture at passage 2, during log-growth and given intravenously. Just before cells are trypsinized, they are rinsed with Tyrode's salt solution then labeled with the vital dye, Dil at a concentration of 1 ug/ml in Tyrode's for 20 minutes at 37°C. Dil was initially diluted to 1 mg/ml in DMSO followed by 1:1000 diluted in Tyrode incubated for 20 minutes at 37°C. Each experimental run used hMSCs from a different donor; 7 different hMSC preparations were used for these studies.

### Bone marrow derived macrophages (BMDM)

BMDM were utilized in a subset of studies (n = 3). Briefly, bone marrow aspirates were obtained from syngeneic mice and cultured in the presence of L929 media as previously defined [21].

### Ceramic Cube Assay

Several *in vitro *assays for bone, cartilage, marrow stroma, fat [22], and an *in vivo *assay [22,23] for the differentiation of bone in porous calcium phosphate ceramic cubes (3 mm) have been used to test for the differentiation potential and purity of the hMSCs [24]. The standard assay "the ceramic cube assay" for this differentiation is the ability of hMSCs to form bone in 3-mm porous calcium phosphate cubes implanted subcutaneously in mice. We have documented that a quantitative assessment of the amount of bone within the pores of the cube is possible [24,25]. The purity of hMSCs was defined as previously published [26,27]. Briefly, cubes measuring approximately 3 mm per side were cut from a cylindrical ceramic rod composed of 40% hydroxyapatite and 60% tricalcium phosphate, generously provided by the Zimmer Corporation. The cubes were washed with deionized water, dried, and then autoclaved. The sterile cubes were immersed in a solution of fibronectin at a concentration of 100 μg/ml. After a partial vacuum was produced by withdrawing air through the cap of the tube with a 30-ml syringe attached to a 22-gauge needle, the cubes were kept in the fibronectin solution for 2 hours, after which they were allowed to dry at room temperature. Human MSCs were trypsinized as described above and resuspended in serum-free medium at a concentration of 5 million cells per ml. We load the cube with a suspension of 5 million cells per ml, estimating the load volume to be 30-40 thousand cells. Fibronectin-coated cubes were added to the cell suspension and a partial vacuum was generated as for the fibronectin coating [28,29]. Cell-loaded cubes were then incubated at 37°C for two hours in a humidified atmosphere consisting of 5% CO_2 _and 95% air. After the incubation period, the cubes were implanted subcutaneously on the dorsal surface of severe compromised immune deficient (SCID) mice. Mice were anesthetized with a rodent cocktail consisting of ketamine, xylazine, and acepromazine as previously described [19]. The skin and subcutaneous tissue at the incision site was injected with Marcaine local anesthetic at a concentration of 0.025%. After the incision was made on the dorsal surface, up to 9 subcutaneous pockets were expanded by blunt dissection. One cube was placed in each pocket, and the incision was then closed with wound clips. Animals were euthanized after 6 weeks and the cubes were fixed with 10% buffered formalin phosphate. The cube score decreases when hMSCs are diluted with human dermal fibroblasts (non-MSCs) which, are used for controls in these studies [20,28]. We have published measurements of the secretion of bioactive factors by hMSCs in growth, osteogenesis and in marrow stromagenesis pathways [30]. Neither the bioactive secretion assays [30] nor the cube scores [20,25] provide potency assays for hMSCs. The cube scores, however, provide the criteria for documenting the differentiation potential of hMSCs [31,32]. Table 1 provides the cube scores for the majority of the hMSC preparations used in experiments reported here, showing that the hMSCs appear to be typical [19].

**Table 1:** Cube Score Evaluation of hMSCs Derived from Human Marrow

hMSC Preparation	Age of Donor	Cell Yield/Plate (× 10^6^)	Cube Score
1444	25	0.7	Not Done

1446	33	0.8	Not Done

1451	29	1.0	1.47

1450	51	0.8	1.29

1553	30	1.19	0.78

1557	47	1.0	1.11

1568	47	0.69	1.12

1594	28	0.79	1.17

1568	45	0.13	1.37

### Cytokine and Inflammatory Mediator Assessment

BAL and serum differentials were measured in duplicate from each individual mouse (no samples were batched) by cytospins and direct cell counts [33]. BAL level of cytokines (R&D Systems, Minneapolis, MN) were assessed by ELISAs and Luminex multiplex assays as previously described [34]. Inter and intra assay variability was controlled through the use of high and low quality control standards and standard curve slope comparison using Statlia Technology (San Diego, CA). The lower range of detection was between 3-10 pg/ml depending on the cytokine in evaluation.

### Statistics

Group and time point comparisons used repeated measures of analysis of variance (ANOVA) and t-tests. Individual significance levels of 0.05 were used for all tests. The designation of "n" in these studies is based upon the number of experimental times the study was performed. In each experiment, there were 4 groups: saline challenged-no hMSCs, saline challenged-hMSCs, ovalbumin challenged-no hMSCs, ovalbumin challenged-hMSCs. Groups were separated into histology/pathology or bronchoalveolar lavage. Each group of histology/pathology had 3-4 mice. Each group of inflammation had 5-7 mice. Statistics were done based upon the total number of experiments including all of the animals in each of the different groups. In some instances a description identifying "n" versus the numbers of animals in each group is explained for clarity.

## Results

### The Asthma Models

**Figure 1:**
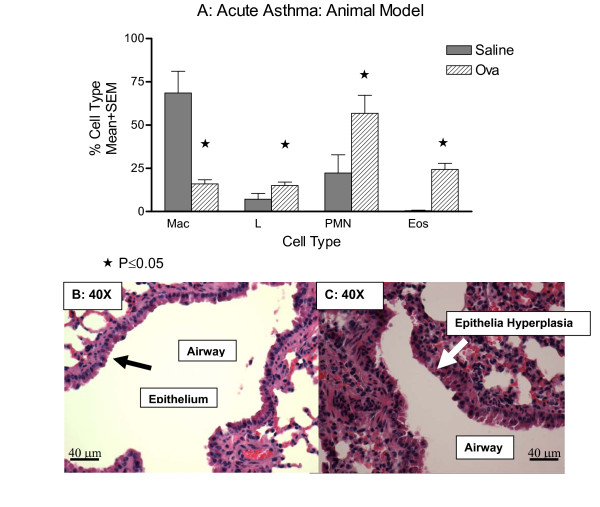
**Establishing the Acute Model of Murine Asthma**. Mice were sensitized with ovalbumin, rested for 14 days and then challenged daily with ovalbumin or sham PBS for 5 days. Mice were sacrificed and the lungs were processed for inflammation with BAL (1A) or histology without BAL (1B saline-challenged (40×) versus 1C ovalbumin-challenged (40×)). Histology is representative of 5 different experiments with 6-8 mice in each group. Inflammation is representative of 5 different experiments with n = 4-6 for each group. Ovalbumin challenged mice had a significant increase in inflammatory cells (n = 4, p = 0.04).

Balb/c mice were sensitized with OVA, and after 14 days the mice were challenged daily for 5 days with either ovalbumin or sham PBS followed by sacrifice for histology or BAL for inflammatory markers. The pulmonary differential of the lungs of ovalbumin-treated mice had significantly more eosinophils (Figure 1) and an increase in total cell count, 25 ± 4% (n = 4, p = 0.04). Evaluation of lung histology in mice sensitized and challenged with saline showed relatively little change in lung morphology (1B) compared with ovalbumin-challenged mice, which showed significant epithelial cell hyperplasia and thickening of the airway consistent with other published observations of this model (1C).

### Localization of Human Mesenchymal Stem Cells

**Figure 2:**
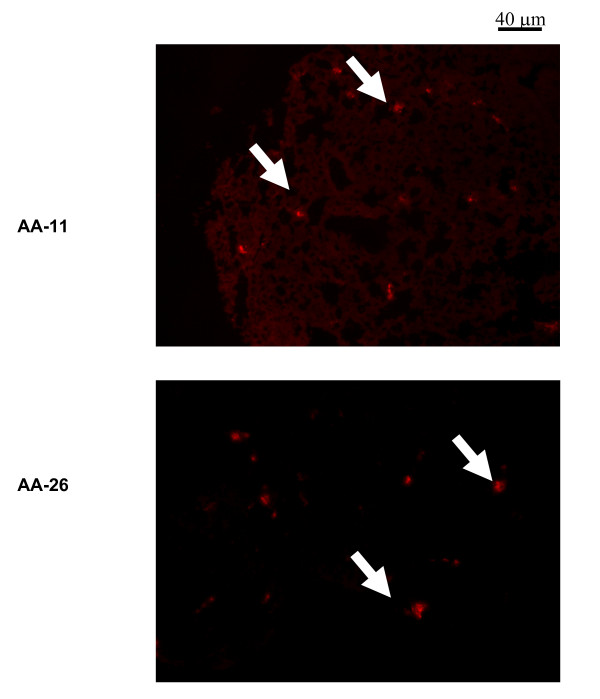
**Dil labeled hMSCs can be Localized in the Murine Lung**. Sensitized and challenged mice from the acute asthma group were given hMSCs 5 days prior to euthanisia. The hMSCs were labeled with the vital dye, Dil. The animals were assessed for the presence of Dil fluorescent hMSCs. Highly positive hMSCs (white arrows) were observed in the lung tissue of mice #11 and #26,.

Localization of hMSCs in the lung suggests a potential therapeutic application to chronic lung diseases such as asthma [13]. To further associate the ability of localizing the hMSCs to the lungs of the mice in the asthma model, hMSCs were labeled with the vital dye, Dil and injected into mice. The animals were euthanized 6 days after intravenous injection of the fluorescent hMSCs. The lungs of the animals were snap frozen and frozen sections were evaluated for the presence of labeled hMSCs. Figure 2 shows an example in which Dil-stained hMSCs were observed in the ovalbumin-challenged animal model, suggesting localization in the lung after intravenous injection. Dil stained hMSCs were also administered to saline challenged mice and evaluated for distribution 6 days post administration. We could detect no labeled hMSCs in the lungs of the saline challenged mice at 6 days. These studies implicate that in inflammation, some of the hMSCs remain in the lung whereas in the absence of inflammation the hMSCs continue elsewhere. Profiling the life of hMSCs *in vivo *in the presence and absence of inflammation is the focus of on-going studies in our laboratory. These results confirm previous observations when using rat models [13].

### Human Stem Cells in Murine Models: Xenographic Effects

**Figure 3:**
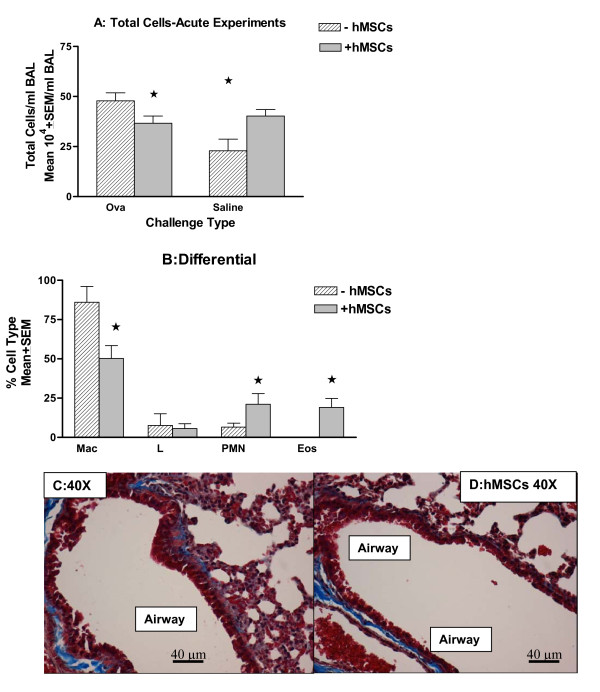
**Xenographic Effect of hMSCs on Total Cell Recruitment**. Animals were sensitized with ovalbumin and challenged with saline followed by BAL. The total differential cell count between saline-challenged mice and naive controls was negligible (data not shown). hMSC exposure decreased the total cell count in the BAL of the ovalbumin-challenged mice (Figure 3A, p = 0.03, n = 5), but increased the total cell count in the saline-challenged mice (Figure 3A, p < 0.05 for the saline control versus saline control with husks). Further, the hMSCs altered the inflammatory phenotype of the saline control animal (3B, n = 4, p < 0.05) suggesting that the hMSCs can induced a cellular response in the absence of ovalbumin-induced inflammation in the context of an acute response. The change in the inflammatory status did not impact the immediate histology of the acute treated model (3C:40× and 3D:hMSC, 40×). Histology is representative of 5 different experiments with n = 6-8 in each group.

hMSCs were introduced by tail vein injection to mice sensitized with ovalbumin and challenged with saline to determine the impact of hMSCs in the absence of established inflammation. These studies focus on defining the impact of using human derived MSCs across species in murine lungs. Figure 3 shows the change in total BAL cell counts between ovalbumin-challenged and saline-challenged mice in the acute model of asthma. Animals with and without intravenous instillation of hMSCs were studied. The differential between the naïve controls and saline-challenged mice is minimal (data not shown). hMSC therapy decreased the total cell recruitment in the context of ovalbumin challenge (Figure 3A, p < 0.05, n = 4) while increasing the total cell recruitment in the acute saline-challenged model in the absence of inflammation (Figure 3A, n = 4, p < 0.05). There was also a change in the inflammatory status of the treated lung as indicated by a decrease in macrophages (p = 0.05) and an increase in neutrophils and eosinophils (Figure 3B, p = 0.04, n = 4). Histologically there was no significant difference between saline-challenged mice with or without hMSCs (compare figure 3C with 3D+MSCs).

### hMSC Effect on Murine Models of Asthma Inflammation

**Figure 4:**
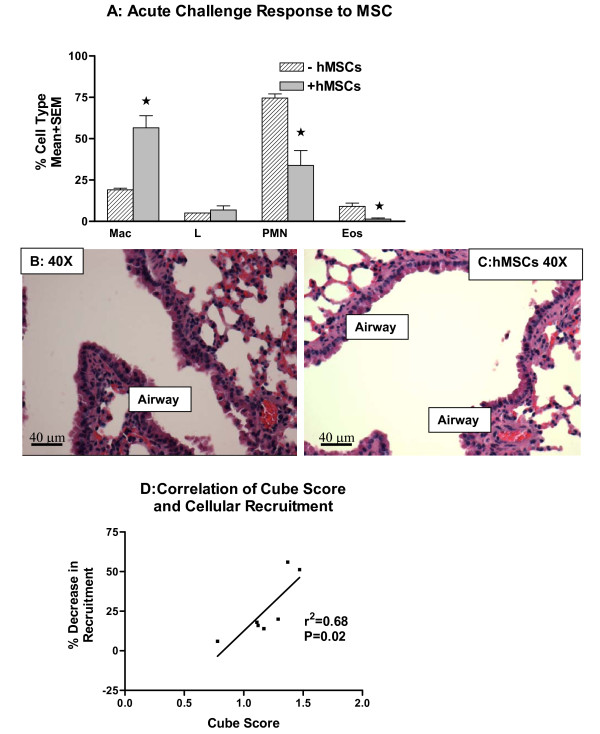
**hMSCs Decrease Acute Inflammation in the Acute Asthma Lung Model**. hMSCs (10^6^/100 ul injected) were given by tail vein injection at day 14 post-sensitization. Mice were evaluated after 5 days of challenge for inflammation. Concurrent mice were evaluated specifically for histology (Figure 4B and 4C). Treatment of the acute asthma mice with MSCs resulted in increased production of macrophages and decreased production of neutrophils and eosinophils. Histologically, the epithelial lining of the bronchiolar airways appears to have less thickening and less surrounding mucus (4C: 40×) when compared with animals not treated with hMSCs (4B: 40×). Histology is representative of 5 different experiments with 6-8 mice in each group. Inflammation is representative of 5 different experiments with n = 4-6 for each group. To determine the relationship between response to hMSCs and the differentiation of the hMSCs, the percent change in total cellular recruitment post-hMSC treatment was plotted against the cube score values in Table I (4D). Cube score values statistically correlated to percent decrease in cellular recruitment (decrease in inflammation) with r^2 ^= 0.68, p = 0.02, n = 7 different cube scores on n = 7 different hMSCs in 7 different ovalbumin challenged asthma experiments.

Mice were sensitized with ovalbumin and rested for 14 days. On day 14, hMSCs were administered to the animals intravenously followed by 5 daily challenges with ovalbumin. The model resulted in increased eosinophils and neutrophils with a decrease in macrophages consistent with the acute asthma murine model (Figure 1). Introduction of hMSCs in this model resulted in a decrease in both neutrophils and eosinophils relative to the ovalbumin-challenged control, and an increase in numbers of macrophages in the BAL fluid of the mice (Figure 4). Histologically, hMSC treatment resulted in a decrease in airway inflammation, mucus production and epithelial cell lining thickening (4C) compared to animals not treated with hMSCs (4B). To further evaluate the relationship between the hMSCs and the inflammation, we correlated the cube score values shown in Table 1 with the overall total cell recruitment in the ovalbumin-challenged model shown in Figure 3. The percent (%) decrease in total cell recruitment in the ovalbumin-challenged asthma model due to hMSC administration was plotted against the cube score values given in Table 1. Figure 4D shows that better differentiation in the ceramic cube assay correlated with *in vivo* decreased BAL total cell counts post-hMSC treatment (r^2 ^= 0.068, p = 0.02). The ceramic cube assay measures osteo-differentiation as a measure of hMSC multipotency. The correlation between immunomodulatory activities *in vivo* provides an alternative means of evaluating *in vivo* potency and efficacy. This is consistent with the observations that the effect of hMSCs via paracrine mechanisms or direct interaction with immune cells, do not depend on cell engraftment and differentiation (35, 36). Studies using a single hMSC preparation in different preparations of the acute asthma model were not done due to the requirement of large numbers, issues of increased passage to generate these numbers, time in culture and other potential environmental induced changes in hMSC phenotype [37-39].

### Administration of hMSCs Alters both Local and Systemic Cytokines

#### Pulmonary Response

**Figure 5:**
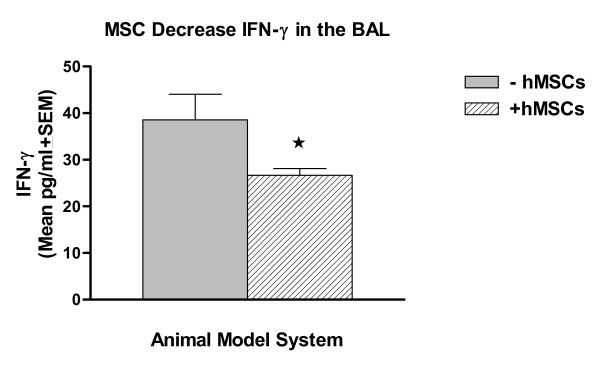
**hMSCs Decrease IFNγ in Acute Asthma**. BAL fluid obtained from mice modeled for acute asthma had significantly less IFNγ when treated with hMSCs *in vivo *(n = 4, p < 0.05).

BAL fluid was obtained from the acute asthma model mice with and without treatment with hMSCs. The lavage fluid was evaluated for IFNγ and essential cytokines of inflammation and activation. The acute asthma model had elevated IFNγ relative to untreated mice which was not detectable (n = 4, p < 0.05), which is consistent with the literature [40]. Treatment of the animals with intravenous hMSCs, resulted in a statistical decrease in BAL IFNγ levels (Figure 5, n = 4, p = 0.05).

#### Systemic Response

**Figure 6:**
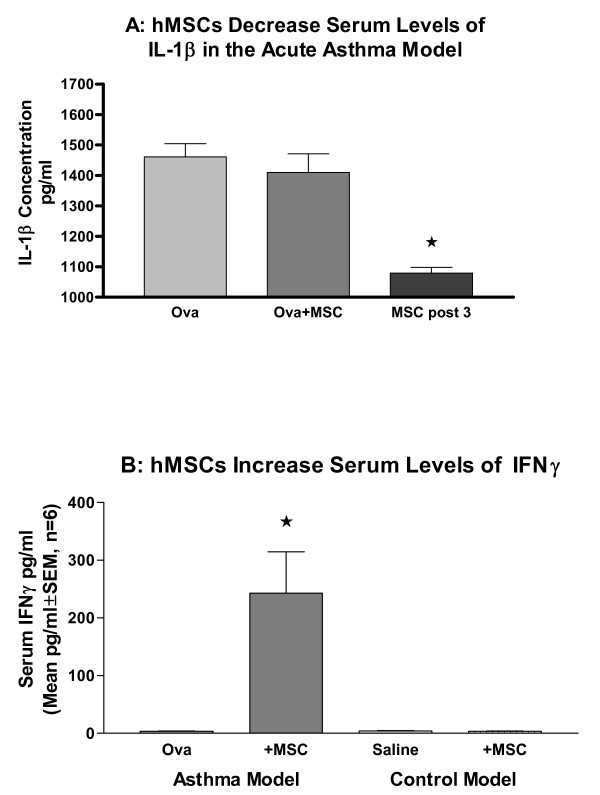
**Serum IL-1β Decreases with hMSC Treatment**. Cardiac puncture was done to obtain adequate serum samples from mice with and without treatment with hMSCs. The acute asthma mice had significantly elevated levels of systemic IL-1β (6A, n = 8, p < 0.001). Systemic IL-1β concentrations were altered by hMSCs in the acute asthma model when hMSCs were given three days after challenge (peak inflammation). hMSCs significantly decreased the level of systemic IL-1β (n = 2, p = 0.05). Systemic IFNγ concentrations were increased in the acute asthma mice after hMSC treatment (6B, n = 4, p = 0.043). This was dependent on the presence of *in vivo *inflammation, since saline animals given hMSCs had no detectable serum IFNγ.

The acute model of asthma had elevated levels of circulating serum IL-1β relative to controls, whose levels were not detectable (Figure 6A, n = 8, p < 0.001). IL-1β levels decreased only if hMSCs were given after initiation of inflammation (hMSCs given post challenge on day 3, p < 0.05), not at the start of the acute challenge. IL-10, IL-5, IL-4 and IL-13 levels were not detectable in the murine model of acute asthma; systemic levels of IFNγ were only observed after treatment with hMSCs (Figure 6B). The IFNγ effect was dependent on the presence of inflammation since saline animals given hMSCs had no detectable circulating IFNγ.

### Dependence of The hMSC Effect on Existing Inflammation

**Figure 7:**
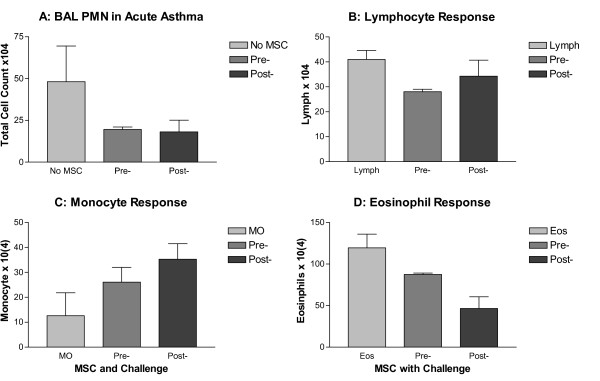
**Lung Inflammation Determines Response to hMSCs**. BAL fluid was obtained from mice treated with hMSCs just prior to challenge and initiation of inflammation (pre-) or 3 days after initiation of inflammation (post-). BAL differentials were obtained from each animal using cytospins and Wright-Giemsa stain. Neutrophil counts are in panel A, lymphocytes in panel B, monocytes in panel C and eosinophils in panel D. The first bar represents the differential in the model without hMSCs. No difference was observed in levels of neutrophils or lymphocytes after hMSC treatment. Pre-challenged mice had a decrease in eosinophils (panel D, n = 4, p = 0.05) compared to control mice. This decrease in eosinophils was amplified at post-treatment with hMSCs. Additionally, post-treatment mice had a significant increase in localized levels of monocyte/macrophages (panel C, n = 2, p = 0.05, 4 animals used in each group for each experiment).

To further investigate the potential of hMSCs to impact inflammation at peak inflammation in the acute asthma model, BAL was performed on mice treated with hMSCs just prior to challenge (day 14) and post-3 days of challenge (day 16) (Figure 7, n = 5). There were no major differences in BAL neutrophil or lymphocyte levels when hMSCs were given either pre-challenge (before inflammation) or 3 days post -challenge (peak inflammation). Pre-challenge mice had an increase in the number of monocytes (C) and a decrease in the number of eosinophils (D, n = 4, p = 0.05) compared to untreated mice. This "effect" of the hMSCs was amplified by giving the hMSCs 3 days post-challenge. While the eosinophils decreased in the lung with the pre-treatment (D) and then with the post day 3 treatment, the monocytes sequentially increase (C). Furthermore, the lungs of the mice given the hMSCs 3 days post-secondary challenge had significantly more monocytes than the untreated animals (n = 4, p = .05, representative of 2 different experiments)

## Discussion

hMSCs have the unique capacity to be both regenerative and serve as conduits of mediators that can immuno-modulate in situations of inflammation. Intravenously injected hMSCs localize in the lung [13] prior to disseminating into the peripheral tissues. Asthma is an inflammatory airway disease characterized by T-cell hyper-reactivity, scarring and remodeling. Since hMSCs have the capacity to inhibit scarring and suppress T-cell activity, we investigated the potential of using hMSCs to reverse airway inflammation in the murine ovalbumin model of acute asthma. Our data show for the first time that hMSCs are well tolerated in the murine model of acute asthma, suggesting that hMSCs can favorably change the outcome of asthmatic inflammation without the pathology associated with cross-species application. Further, our data show that hMSCs given after the induction of airway disease dramatically reverse the airway inflammation associated with the ovalbumin model of acute asthma. Additionally, the short-term nature of the acute asthma model and the *in vivo *responsiveness to hMSCs suggest that the acute model can be used to measure hMSC effectiveness *in vivo *with the correlation of the standard cube score with percent decrease in lung cell recruitment in response to antigenic challenge. The ceramic cube assay measures osteo-differentiation as a measure of hMSC multipotency and has, historically served as the gold standard. The correlation between immunomodulatory activities *in vivo* provides an alternative means of evaluating *in vivo* potency and efficacy. This is consistent with the observations that the effect of MSCs via paracrine mechanisms or direct interaction with immune cells, do not depend on cell engraftment and differentiation [35,36]. Future studies will include hMSC dose-response and different modes of administration.

Adult hMSCs isolated from bone marrow are able to differentiate in culture into a number of mesenchymal phenotypes including those that form bone, cartilage, muscle, fat and other connective tissues [23]. Originally, it has been suggested that hMSCs are responsible for the normal turnover and maintenance of adult mesenchymal tissues. More recently, hMSCs have been shown to reside in a number of tissues as pericytes, suggesting that they can have a major impact on focal injuries [1,2,41]. If hMSC are capable of impacting the local milieu, they could be used therapeutically as allogeneic sources of repair *in vivo *[42]. The therapeutic implication of hMSCs is based upon the observation that culture-expanded hMSCs have no detectable MHC class II cell surface markers or co-stimulator molecules [4], suggesting that the hMSCs evade immune surveillance by the host. In our model, hMSCs induce white blood cell recruitment in the acute model when the animals lack the inflammation associated with the secondary challenge with ovalbumin. Although the BAL differential and the total cell counts were altered by hMSCs in the acute control, no detectable changes in lung histology are detected, suggesting the absence of adverse response of the host tissue to the hMSCs in the saline-challenged acute model.

hMSCs were first used to supplement bone marrow transplantations because hMSCs were assumed to home back to the bone marrow stroma and have the potential to efficiently prefabricate the injured marrow stroma for human stem cell engraftment and subsequent hematopoietic lineage functions [10]. Early successful clinical trials supported the idea that culture-expanded hMSCs were, indeed, capable of promoting successful engraftment of hematopoietic progenitors and their production of circulating mature blood cells efficiently and safely [43,44]. Allogeneic engraftment has also been used to treat gene defects [45,46]. In these cases, culture-expanded hMSCs from the allo-donor were used to supplement the bone marrow transplantation of host with defective genotypes with the assumption that the hMSCs homed to marrow and re-established the stroma to enhance allogeneic engraftment. For example, Horwitz and colleagues reported improvement in six children with osteogenesis imperfecta treated with allogeneic bone marrow transplantation [45]. These studies used allogeneic hMSCs with no adverse events. In studies using the bleomycin model, administration of hMSCs into mice immediately after exposure to bleomycin was associated with a significant reduction in inflammation and collagen deposition associated with the lung disease [47,48]. In these studies, the rates of engraftment were undetectable or at the limits of detection. The implication is that the mechanism of the hMSC effect and improvement in the bleomycin- induced inflammation was not particularly due to stem cell engraftment of the injured tissue, but to the effects of paracrine secretion of growth factors and cytokines which stimulate repair.

hMSCs have been shown to effectively shut down graft versus host disease (GvDH), a T -cell mismatched immune-mediated disease [49]. Osiris Therapeutics http://www.osiristx.com has documented that during a study of compassionate use of adult marrow-derived culture-expanded allogeneic hMSCs in children with steroid resistant GvHD, 7 out of 12 had complete remission of GvHD at one month and 95% were alive at 6 months. In addition, 9 out of 12 had complete recovery from their gastrointestinal GvHD, and the remaining 3 had their severity reduced to Grade I gastrointestinal GvHD. The allogeneic MSCs in these studies, even with the multiple infusions, induced no adverse events http://www.osiristx.com. This is consistent with our observations using hMSCs in the saline sensitized, saline-challenged control mice. These mice had no change in lung pathology and minimal change in inflammatory status as defined by BAL differential. These and other studies established that isolation and culture expansion is safe with clinical benefit from the intravenous delivery of allogeneic hMSCs. These observations suggest that the trophic effects of hMSCs played a profound role in the observed therapeutic benefit in all of the above studies.

In terms of a potential therapeutic, our data show that hMSCs are effective at attenuating or reversing the inflammation and pathology associated with acute asthma in the ovalbumin murine model of this airway disease. The acute model of murine asthma generated increased pulmonary inflammation and histology consistent with increased mucus production and eosinophils. hMSC treatment of acute asthma mice resulted in decreased airway inflammation as determined by BAL. Histologically, the airways showed signs of decreased epithelial lining thickening and mucus hyper-production. hMSCs in culture secrete a variety of molecules, both bioactive and extracellular matrix, in response to their local environment and their activity status [7,8,50]. In our studies, not only did hMSC in children decrease the pathology and inflammation in the acute murine asthma, but there was a significant decrease in lung IFNγ levels consistent with decreased immune activation. Systemically, administration of hMSCs increased IFNγ, while at the same time decreased IL-1β. These studies suggest a mechanism by which IFNγ/IL-1β levels may alter the host inflammatory response in an allergic setting through altering systemic cytokines. Unlike other models [35], we did not detect IL-10, IL-5, IL-4 or IL-13 in the BAL fluid from our model, which may be due to tissue localization instead of soluble secretion into the BAL fluid. The differences may also be due to the model of selection. This is the focus of on-going research and future manuscripts. In addition to understanding how hMSCs regulate *in vivo *systemic and localized IFNγ/IL-1β levels, our focus will also include factors secreted by the hMSCs or by the host in response to MSCs, which alter the course of asthma. Some possible candidates include TGS-6, SDF-1, MPC-3 and Hif-1α [51,52].

Aerosolized corticosteroids are the standard anti-inflammatory medication for asthmatics and are effective in most cases. However, a substantial number of asthmatics remain symptomatic even with the addition of steroid-saving long-acting beta[2]-adrenoreceptor agonists [53]. Variability in immune challenge and response to therapy makes asthma a difficult disease to monitor and manage. Further, long term treatment of patients with systemic corticosteroids for severe asthma raises concerns due to the secondary effects of the therapeutic. New treatment options targeting the pathophysiologic events causing development and persistence of asthma are desired for these patients [54]. The presentation of hMSCs limits the field of damage or injury and inhibits fibrosis or scarring at sites of injury in the ovalbumin model of asthma. The infused hMSCs secrete immuno-modulatory agents which deactivate T-cell surveillance and chronic inflammatory processes [8]; thus, allogeneic or xenogeneic MSCs can be therapeutically effective. The data presented in this manuscript implicate the trophic products of hMSCs in attenuating inflammation in the acute asthma murine model. Additionally, the acute asthma model of inflammation may be a viable option for gauging hMSC efficacy and function *in vivo* in conjuction with *in vitro *assays such as the ceramic cube model.

## Competing interests

The authors declare that they have no competing interests.

## Authors' contributions

TB directed, analyzed and planned all of the studies. MN carried out the studies and helped with the methods. DL isolated the hMSCs, did the cube scores and provided information on the technology associated with the hMSCs. AC provided insight on the hMSCs impact on the asthma model and the identification of the potential for hMSC efficacy testing. AC laboratory provided the support for the stem cell production, validation and application. All authors read and approved the final manuscript.
